# Reducing PICU-to-Floor Time-to-Transfer Decision in Critically Ill Bronchiolitis Patients using Quality Improvement Methodology

**DOI:** 10.1097/pq9.0000000000000506

**Published:** 2022-01-21

**Authors:** Cristin Q. Fritz, Blake Martin, Megan Riccolo, Michelle Fennell, Elise Rolison, Todd Carpenter, Lalit Bajaj, Amy Tyler, Mark Brittan

**Affiliations:** From the * Children’s Hospital Colorado, Aurora, Colo.; † University of Colorado School of Medicine, Aurora, Colo.; ‡ Adult and Child Consortium for Health Outcomes Research and Delivery Science, University of Colorado, Aurora, Colo.

## Abstract

**Introduction:**

Specific criteria for de-escalation from the PICU are often not included in viral bronchiolitis institutional pathways. Variability of transfer preferences can prolong PICU length of stay. We aimed to decrease the time from reaching floor-appropriate heated high flow nasal cannula (HHF) settings to the transfer decision by 20% through standardizing PICU-to-floor transfer assessment in a PICU bronchiolitis cohort.

**Methods:**

We included PICU bronchiolitis admissions from October 2019 to April 2020, who were 6-months to 2-years-old with no comorbidities nor intubation during their encounter. Our intervention bundle included introduction of transfer criteria and standardization of transfer-readiness assessment. The primary outcome was time from reaching floor-appropriate HHF settings [8 L per minutes (Lpm)] to placement of the transfer order (“time-to-transfer decision”). The secondary outcome was PICU length of stay. The main process measure was the proportion of patients transferred on ≥6 Lpm HHF. Balancing measures included Rapid Response Team activation and unplanned PICU readmission. We assessed admissions meeting inclusion criteria from December, 2018-March, 2019 for the preintervention baseline.

**Results:**

Special cause variation indicated improvement in our primary outcome and process measures. Comparison of baseline to postintervention revealed a reduction in median time-to-transfer decision (14.4–7.8 hours; *P* < 0.001) and increase in children transferred on ≥6 Lpm (51%–72%; *P* < 0.001). We observed no change in PICU length of stay or balancing measures.

**Conclusion:**

Standardizing de-escalation criteria and transfer-readiness assessment reduced the time-to-transfer decision out of the PICU and increased the proportion transferred on ≥6 Lpm HHF for children with viral bronchiolitis without increasing PICU readmissions.

## Abstract 2

Supplemental Digital Content is available in the text.

## INTRODUCTION

Delay in transfer from the Intensive Care Unit (ICU) to the floor has significant implications for hospital and patient outcomes. Transfer delays can contribute to unnecessary ICU time, canceled elective surgeries, and decreased hospital throughput.^1,2^ ICU bed strain and delayed transfer are associated with worse patient outcomes^3^ and decreased patient satisfaction.^4^ Both the pediatric and adult literature identify operational contributors to delayed transfer out of the ICU, including appropriate bed availability and poor communication between medical teams.^1,4–12^ Prior improvement work has targeted these downstream, operations-related drivers of a delayed transfer.^4–6,8,11,12^ However, identifying patients as medically ready for transfer is a critical component that has received less attention.

Viral bronchiolitis is one of the most common reasons for Pediatric ICU (PICU) admission.^13^ Although institutional guidelines for escalation of care to the PICU are common, standardized, clearly-defined criteria for de-escalation from the PICU to the floor are not often included in institutional pathways. Lack of clear guidance can lead to variable transfer practices and delays in identifying transfer-ready patients. There are no reports of structured improvement efforts that target this aspect of care.

The overall goal of our project was to standardize PICU-to-floor transfer assessment in a subset of patients admitted for viral bronchiolitis. We theorized that providing clear transfer criteria and a structured process for identifying transfer-ready patients would empower care providers and facilitate the timely transfer. Our specific aim was to decrease the time to provider-initiation of the transfer process after patients reached floor-appropriate heated high flow nasal cannula (HHF) settings by 20% by May 2020. Our secondary aim was to reduce PICU Length of Stay (LOS) by 20% during the same timeframe.

## METHODS

### Context

This Quality Improvement (QI) initiative took place in a large, tertiary care pediatric hospital serving a seven-state catchment area in the West. There are 48 PICU beds and 120 general inpatient beds. The PICU cares for greater than 3000 noncardiac admissions per year. Patients with bronchiolitis account for ~18% of all PICU admissions. The Hospital Medicine service is the primary floor service for patients with bronchiolitis. Per hospital policy, the maximum HHF rate allowed on the inpatient floor for patients 3-months-old to 2-years-old is 8 Liters per minute (Lpm).

When the PICU team determines a patient is medically ready for transfer, the provider places a “Decision-to-Transfer” order to initiate the bed assignment process. Once assigned, the provider and nurse give verbal sign-out to the accepting floor team, followed by the provider placing a “Transfer Now” order to initiate the physical transfer. The PICU attempts to minimize floor transfers between 10 pm and 6 am unless unit occupancy is 85% of maximum bed capacity or greater.

### Improvement Team

Our core multidisciplinary improvement team consisted of PICU and Hospital Medicine fellows and faculty, floor nursing leadership, a Process Improvement specialist, and QI faculty. In addition, the core team engaged additional key stakeholders throughout the process, including PICU and Hospital Medicine attending physicians, Advanced Practice Providers, nursing leaders, registered nurses, respiratory therapists, and pediatric residents that served as clinical champions.

### Designing the Intervention

Initial interviews with 30 Hospital Medicine and PICU providers, nurses, and respiratory therapists revealed an existing transfer process subject to variable provider practice patterns, an unclear communication process, and uncertainty regarding floor care team scope of practice. For example, many PICU providers assumed that floor providers and nurses would only accept patients from the PICU if they had a “buffer” in HHF rate below the floor-allowed maximum to allow for subsequent escalation; however, the floor providers did not identify this as a requirement. The improvement team conducted chart reviews on a subset of 50 patients with bronchiolitis admitted to the PICU during the 2018–2019 bronchiolitis season to validate this anecdotal evidence. Time-to-transfer decisions ranged from 3 to 43 hours after a patient reached floor-appropriate HHF settings. Although some variations may have resulted from non-respiratory patient factors or floor bed availability, the extensive range suggested a contribution from variation in transfer practices.

**Fig. 1.**
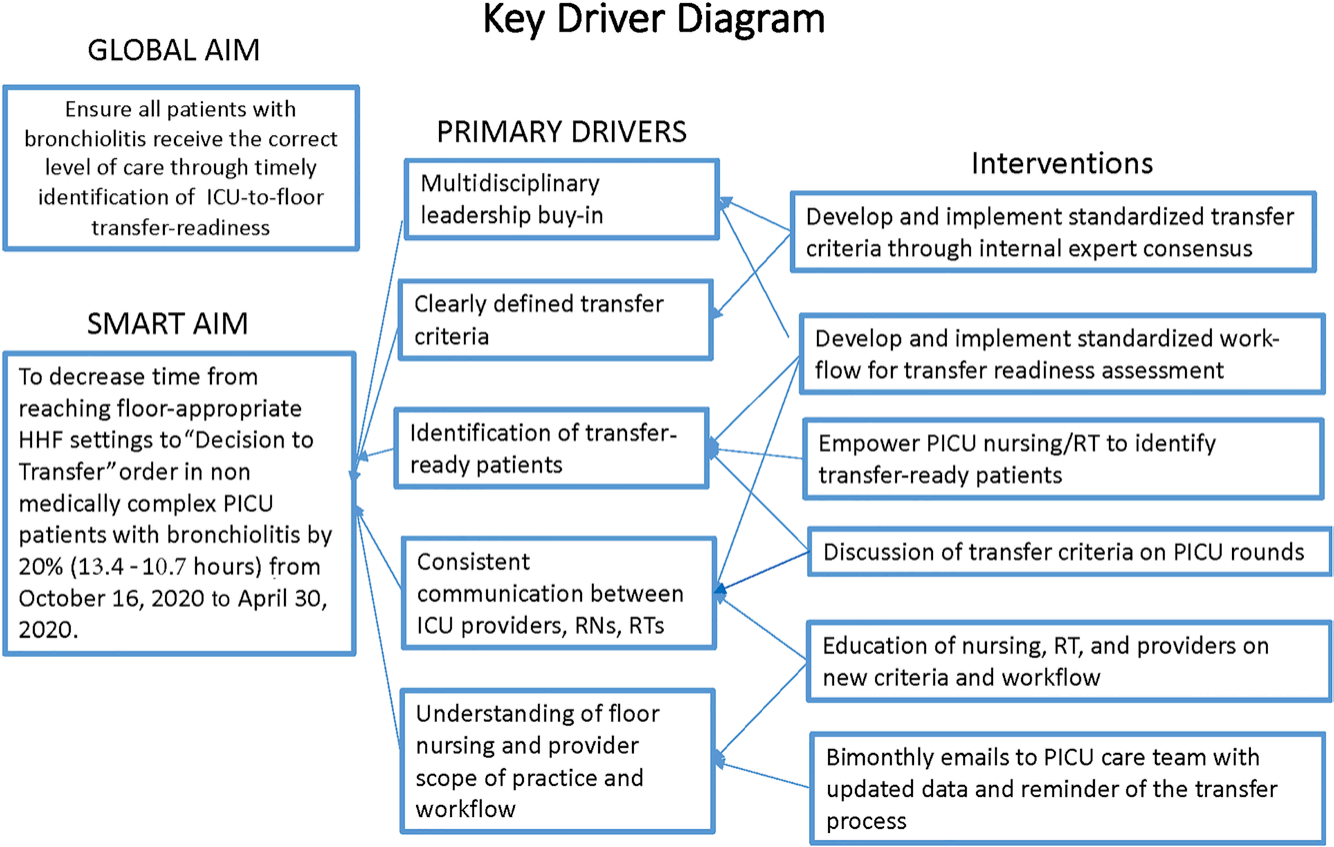
Key Driver Diagram. RT, respiratory therapy.

We created a current-state process map for the transfer process (**See figure, Supplemental Digital Content 1**, which shows pre-intervention state process map, http://links.lww.com/PQ9/A338) to identify targets for improvement. We also assessed barriers to consistent transfer practices, which included lack of provider consensus on floor-readiness, lack of a systematic process for identifying transfer-ready patients, inaccurate assumptions around floor nursing scope of practice, inefficient communication among PICU care providers regarding the current level of HHF support, lack of specific guidelines for HHF rates at transfer, and need for multidisciplinary leadership buy-in for change (Fig. 1).

This project was reviewed and approved by the Organizational Research Risk and Quality Improvement Review Panel, the institution’s QI oversight body. We also secured executive sponsorship from the institution’s Chief Medical and Chief Safety Officers.

### Improvement Activities

Our team developed interventions consisting of specific transfer criteria, a standardized process for transfer readiness identification, and leadership engagement to address the identified key drivers.

### Intervention Inclusion/Exclusion Criteria

We included patients 6- to 24-months-old with a primary diagnosis of bronchiolitis, no history of intubation during their admission, and no underlying comorbidities (which might predispose to more serious illness). These criteria were chosen through expert consensus to identify an “otherwise healthy” cohort at a lower risk for disease re-escalation. Of note, in our preintervention bronchiolitis sample, 13 of 50 (26%) children would have been excluded using these criteria. Concomitant community-acquired pneumonia and acute bronchospasm requiring intermittent albuterol were not exclusion criteria (Table 1). The admitting provider determined whether a patient qualified for inclusion based upon these criteria.

**Table 1.** New Transfer Pathway Inclusion and Exclusion Criteria along with Corresponding Data Source(S) Used

Inclusion Criteria	Data Source(s)
Age 6 months–2 years	Birth date
Primary diagnosis of bronchiolitis	Problem list
**Exclusion Criteria**	**Data Source(s**)
Cardiovascular disease (*eg, Congenital Heart Disease, Congestive Heart Failure, Pulmonary Hypertension*)	Problem list, provider documentation
Chronic lung disease (*eg, Baseline respiratory support, Bronchopulmonary Dysplasia, Persistent asthma*)	Problem list, provider documentation
Neurologic or neuromuscular disease (*eg, Cerebral palsy, Dysphagia,Epilepsy, Traumatic brain injury,Significant developmental delay and/or hypotonia (eg Trisomy 21), Ventriculoperitoneal shunt*)	Problem list, provider documentation
Gestational age younger than 36 weeks	Problem list, provider notes
Intubation during admission	PICU provider notes, order history, lines/drains/airway documentation
Acute bronchospasm *Only if requiring continuous albuterol*	Problem list, PICU provider notes, medication history
*Patients not excluded for:	**Data Source(s**)
Community acquired pneumonia *Treated with antibiotics*	PICU provider notes, radiology reports, medication history
Acute bronchospasm *Requiring intermittent albuterol and/or steroids*	Problem list, PICU provider notes, medication history

### Transfer Criteria

We developed an initial set of transfer criteria to optimally balance patient safety with efficient patient flow. We considered patients candidates for transfer if they remained stable on 8 Lpm HHF or greater for 6 hours and required suctioning less than every 2 hours.

### Transfer-readiness Identification Process

**Fig. 2.**
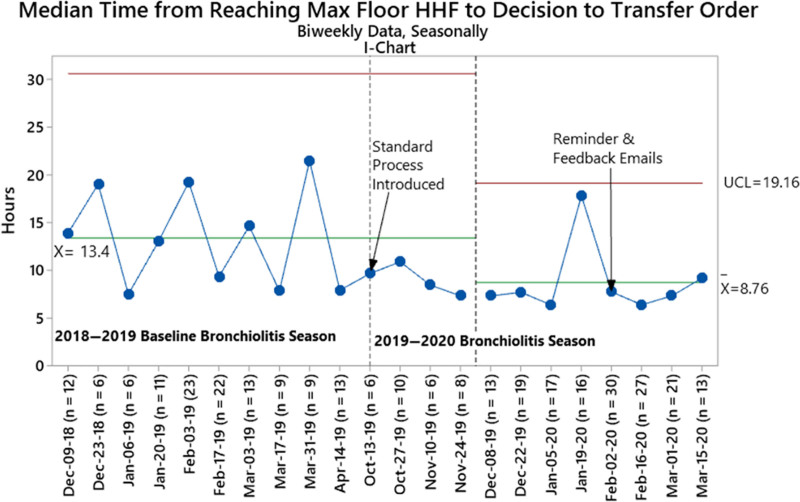
Median time from reaching maximum allowable floor HHF settings to the placement of the Decision-To-Transfer order during baseline and postintervention periods. Baseline centerline extended into the improvement period until special cause met on 1/5/20 (2 of 3 points >2 standard deviations from centerline), shifted at the first point. Bi-weekly data were combined with consecutive data points due to a sample size of less than 5 for the first timeframe in December 2018 and final timeframes in April 2019 and March 2020. Medians in pre-post analyses differ from control chart centerlines because the latter represent the average of biweekly medians. UC, upper control limit.

Our team created a future-state process map outlining the new transfer readiness identification process (**See figure 2, Supplemental Digital Content 2,** which shows future state process map, http://links.lww.com/PQ9/A339). Upon admission, the PICU nurse and provider discuss if the patient meets inclusion criteria. The PICU nurse then contacts the patient’s provider upon reaching 8 Lpm HHF to communicate that the 6-hour observation period has started. If the patient does not re-escalate in required respiratory support during this period, the provider clinically assesses the patient’s transfer-readiness. If deemed not ready for transfer, the nurse documents that the patient is no longer on this pathway. If deemed clinically appropriate for transfer, the provider enters the Decision-to-Transfer order. The proposed transfer pathway was iteratively reviewed with key stakeholders before tests of change and socialized in multiple venues to reach all involved personnel. Before our intervention, PICU providers often identified bronchiolitis patients as medically ready for transfer during morning rounds. The new process encouraged transfer at any point that the child met criteria other than 10 pm–6 am if unit occupancy was less than 85% of maximum bed capacity (per PICU unit policy).

### Initial Project Implementation (October 2019; Plan-Do-Study-Act Cycle 1)

**Fig. 3.**
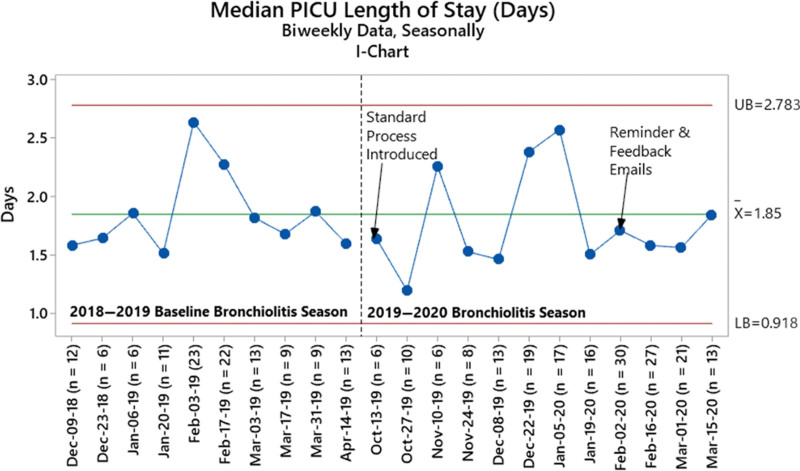
Median PICU length of stay during baseline and postintervention periods. The baseline centerline extended into the improvement period. Biweekly data were combined with consecutive data points due to sample size of less than 5 patients for the first timeframe in December 2018 and final timeframes in April 2019 and March 2020. Medians in pre-post analyses differ from control chart centerlines because the latter represent the average of biweekly medians. LB, lower bound; UB, upper bound.

Project members disseminated information regarding the new process to their department via email, leadership meetings, and departmental meetings. We placed educational flyers with inclusion and exclusion criteria throughout PICU and floor workspaces to aid staff in identifying qualifying PICU patients (**See figure 3, Supplemental Digital Content 3,** which shows educational flyer. Copies of the flyer were placed throughout the PICU and floor workspaces prior to project implementation to aid staff in identifying qualifying patients, http://links.lww.com/PQ9/A340). Project members debriefed with PICU and floor teams after rounds and provided reminders about the new process. Education occurred monthly with residents at the start of their PICU rotation. Nursing leadership included conversations about the new process in daily huddles and the PICU newsletter.

### Twice Monthly Data Updates (February 2020; Plan-Do-Study-Act Cycle 2)

**Fig. 4.**
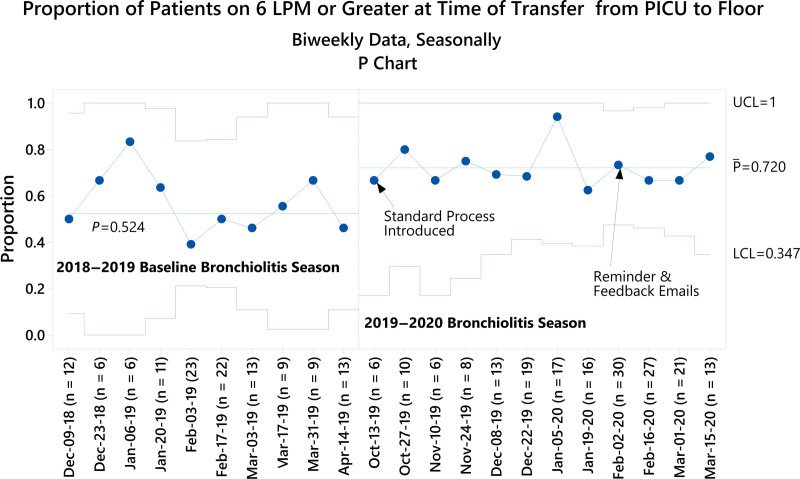
The proportion of patients transferred on 6 Lpm HHF or greater during baseline and postintervention periods. Baseline centerline extended into improvement period until special cause met on 1/19/20 (8 points above centerline), at which point the centerline was shifted at the first point (10/13/2019). Biweekly data were combined with consecutive data points due to a sample size of less than 5 patients during the first timeframe in December 2018 and final timeframes of April 2019 and March 2020. Medians in pre-post analyses differ from control chart centerlines because the latter represent the average of biweekly medians. LCL, lower control limit; UCL, upper control limit.

To maintain project visibility, we sent bimonthly emails to PICU staff with reminders about project aims, transfer criteria, and interim data updates to demonstrate our progress. (**See figure 4, Supplemental Digital Content 4,** which shows the complete project timeline. http://links.lww.com/PQ9/A341).

### Study of Interventions

#### Measures

The primary outcome was the median time in hours from a patient reaching the maximum floor-appropriate HHF settings (8 Lpm) to the placement of the “Decision-to-Transfer” order (“time-to-transfer decision”). If a patient re-escalated during the 6-hour observation window, they were taken off the pathway until they were again weaned to 8 Lpm. We chose the “Decision-to-Transfer” order as our endpoint because it serves as a proxy for medical readiness for transfer. Ideally, operational factors outside this project’s scope should not be impacted (eg, floor bed availability). The secondary outcome was median PICU LOS. The initial process measure we proposed was nursing documentation of the 6-hour provider assessment decision. However, this documentation was rarely performed. Therefore, the substitute process measure we used was the proportion of patients transferred to the floor receiving 6 Lpm or greater. We chose this value to serve as a proxy for the proportion of patients transferring near their maximum HHF floor rate of 8 Lpm and, presumably, earlier in their clinical course. Balancing measures included Rapid Response Team (RRT) activations and unplanned PICU readmissions within 24 hours of transfer among included children.

#### Data Collection and Analysis

Through chart review, we obtained clinical characteristics and outcomes data for the baseline (December 2018–March 2019) and postintervention (October 2019–April 2020) periods. In addition, during the postintervention phase, PICU and Hospital Medicine team members reviewed PICU-to-floor bronchiolitis patient transfers weekly to identify patients meeting inclusion criteria.

We analyzed outcome and process measures using statistical process-control charts, including biweekly data from the baseline and the postintervention seasons. To measure the median time-to-transfer decision and PICU LOS, we constructed I-charts, where the centerline averages bi-weekly medians. We created a P-chart to measure the proportion of children transferred to the floor receiving 6 Lpm or greater. Biweekly data points with less than 5 patients were combined with consecutive data points. We used special cause variation rules outlined by Carey et al.^14^ to identify a statistically significant change in the processes. (**See document, Supplemental Digital Content 5,** which shows the supplemental methods. http://links.lww.com/PQ9/A342)

We compared clinical characteristics and outcome measures between baseline and postintervention periods using Fisher’s Exact Test for categorical variables and Mann-Whitney U tests for continuous variables. To determine intervention effect on the various phases of a patient’s hospitalization (eg, time in PICU versus on the floor), we compared the median time spent in each of four phases of care (**See figure 5, Supplemental Digital Content 6,** which shows median number of hours spent in each phase of a patient’s hospitalization. Time on floor prior to PICU admission not shown. Note that because each phase value listed is the median value for that specific phase over all patients that the sum of phases 1, 2, and 3 does not equal the median PICU LOS, http://links.lww.com/PQ9/A343) between the two groups. We performed all data analysis using R version 3.5.3 (R Foundation, Vienna, Austria). All statistical tests were performed with a level of significance of α = 0.05.

## RESULTS

During the postintervention period, 189 of 461 (41%) patients admitted to the PICU for bronchiolitis met inclusion criteria. The most common reasons for exclusion were age younger than 6 months (39%), prematurity (18%), and receiving continuous albuterol (15%). There were no differences in age or proportion of patients treated for pneumonia before and after the intervention among included patients. Still, a higher percentage of patients were treated for acute bronchospasm during the postintervention period (Table 2).

**Table 2.** Baseline and Postintervention Cohort Patient Characteristics and Outcomes

Category	Baseline (N = 123)	Postintervention (N = 189)	*P*
Patient characteristics			
Age (y)	1.1 (0.8–1.5)	1.2 (0.8–1.5)	0.36
Acute bronchospasm treated in PICU	27 (22)	63 (33)	0.03
Pneumonia treated in PICU	31 (25)	44 (23)	0.79
Outcome and process measures			
Time-to-transfer (h)	14.4 (6.8–22.9)	7.8 (6.1–16.8)	<0.001
PICU LOS (d)	1.82 (1.47–2.62)	1.75 (1.25–2.54)	0.15
Patients transferred on ≥6L HHF	63 (51)	137 (72)	<0.001
Balancing measures			
Rapid Response Team activation*	2 (1.6)	3 (1.6)	1.0
Unplanned PICU readmission*	1 (0.8)	2 (1.1)	1.0

*Within 24 hours of transfer to the floor.

Results presented as median (IQR) for continuous variables and n (%) for categorical variables.

We identified special cause variation on the median time-to-transfer decision I-chart during the postintervention period signaling an improvement in our primary outcome (13.4 hours preintervention to 8.8 hours postintervention, calculated via an average of biweekly medians) (Fig. 2). Our intervention led to a decrease in the overall median time-to-transfer decision as well from 14.4 hours (IQR 6.8–22.9) at baseline to 7.8 hours (6.1–16.8) postintervention (*P* < 0.001) (Table 2). The higher value observed on 1/29/2020 may be related to the high hospital census as several transfers were delayed while awaiting an available floor bed.

We observed no difference in our secondary outcome of median PICU LOS from 1.82 days (IQR 1.47–2.62) at baseline to 1.75 days (IQR 1.25–2.54) postintervention (*P* = 0.15) (Table 2, Fig. 3). Time from PICU admission to reaching max floor HHF settings was similar between the baseline and postintervention groups, as was time from the Decision-to-Transfer order to physical transfer to the floor (**See figure 5, Supplemental Digital Content 6,** which shows…http://links.lww.com/PQ9/A343).

Finally, we identified special cause on the P-chart for the proportion of patients transferred receiving 6 Lpm HHF or greater, the primary process measure used, demonstrating improvement (Fig. 4). The percent of patients transferred on 6 Lpm or greater increased from 51% at baseline to 72% postintervention (*P* < 0.001) (Table 2).

Comparing baseline to the postintervention period, there was no change in rate of RRT activation (1.6% versus 1.6%; *P* = 1.0) or rate of PICU readmission within 24 hours of transfer (0.8% versus 1.1%; *P* = 1.0).

## DISCUSSION

The lack of defined medical criteria for care de-escalation from the PICU to the floor among patients with bronchiolitis can delay the transfer of floor-appropriate patients. As demonstrated in White et al,^15^ standardized criteria for de-escalation of care for patients with common pediatric conditions can safely improve LOS. This study developed standardized criteria and a structured process for identifying transfer-ready patients with bronchiolitis in our PICU. After implementing these criteria and the new process, we observed a significant 46% decrease in the time-to-transfer decision and an increase in the proportion of patients transferred on 6 Lpm HHF or greater without significantly increasing RRT activation or PICU readmission.

Prior improvement work focused on decreasing PICU LOS has targeted operational processes that delay the transfer, such as poor communication between PICU and floor teams and floor bed availability.^8,9,11^ Our project is the first that primarily sought to impact the identification of medical readiness for transfer. As a result, we witnessed rapid adoption of our new transfer process and criteria among PICU providers, which was reflected in our primary outcome (Fig. 2). Additionally, this change was sustained throughout the season except for 2 weeks in mid-January (which may have resulted from limited floor bed availability).

The effect of the intervention on our secondary outcome, PICU LOS, was less dramatic. Given that we observed a reduced time to recognize floor readiness (as demonstrated by our primary outcome), it is surprising that PICU LOS was not reduced. It is possible that other factors (such as limited overnight HHF weaning, hospital nursing and bed shortages, the timing of provider and nursing handoff, and unit practice to limit overnight transfers unless at ≥85% capacity) may have obscured the expected reduction in PICU LOS [32/189 (17%) of postintervention patients met criteria for transfer during the overnight 10 pm–6 am window, for example]. Further study is needed to optimally translate our intervention into a more significant reduction in PICU LOS.

We were also able to increase the proportion of patients transferred near their floor maximum HHF. Floor nurses and providers managed these higher acuity patients without issue and appreciated the opportunity to operate near the top of their scope of practice. Notably, the project did not result in a significant increase in RRT activation or PICU readmission. This finding supports 6 hours of observation on floor-appropriate HHF settings as an initial safe and actionable transfer criterion that others can adapt through additional tests of change.

The success of our improvement work was primarily due to early and frequent socialization of the new process, broad leadership buy-in, and empowerment of all care-team members to be proactively involved in the de-escalation process. In addition to these successes, there were also unexpected consequences. Primarily, the 6-hour observation period became so ingrained in the culture of the PICU that there were attempts to apply it to patients outside the intended cohort. Ongoing education on the details and scope of our project was required to address this challenge.

### Limitations

Several limitations should be considered when interpreting the results of this QI work. First, nursing documentation of provider assessment of the patient after the 6-hour observation period was inconsistent. In cases of delayed transfer, this made it challenging to distinguish between patients who were assessed at 6 hours but not medically ready and those where the transfer pathway was not followed. This lack of assessment documentation also made measurement of associated process measures challenging. Specifically, we could not measure the percentage of cases in which the provider assessed the patient at the 6-hour mark. We instead measured the proportion of patients being transferred to the floor receiving 6 Lpm or greater, using this as a surrogate for the frequency of transfer (and presumably assessment) occurring earlier in a patient’s PICU course. Qualitatively, however, project champions observed, and PICU team members reported that the 6-hour assessment was regularly taking place. In summation, though introducing a new documentation process for nurses to confirm the 6-hour assessments was unsuccessful, PICU providers confirmed that the transfer guidelines were being consistently applied and the 6-hour assessments were occurring.

Second, unit practice limiting overnight patient transfer (unless near capacity) may have increased time-to-transfer decisions for a subset of patients, potentially decreasing the observed effect of our intervention. Third, these interventions took place at a tertiary care children’s hospital with robust QI infrastructure and focus on bronchiolitis QI initiatives, which may limit generalizability. Fourth, variation in hospital policies guiding the degree of respiratory support allowed on the floor may also limit applying the specific criteria developed for this project. However, other institutions can utilize our template to standardize de-escalation and transfer criteria to align with their local practices. Lastly, the COVID-19 pandemic impacted our project by abruptly decreasing both overall and bronchiolitis-specific PICU admissions. This changed hospital transfer policies and prevented subsequent Plan-Do-Study-Act cycles due to a shift in hospital focus and resources to responding to the pandemic.

### Next Steps

During the 2020–2021 respiratory season, we are expanding the age range to include children as young as 3 months of age (which would have allowed for an additional 43 children during the 2019–2020 season). In addition, the team is planning tests of change to increase appropriate overnight transfers, spread the process to affiliated sites in the institution’s Network of Care, and develop an EMR-driven, automated identification process of patients ready for transfer assessment. We also intend to gather family feedback on the current process and proposed future tests of change. Finally, we would ultimately like to expand on the principles developed in this project to create standardized de-escalation transfer criteria for other common PICU diagnoses.

## CONCLUDING SUMMARY

Novel introduction of standardized transfer criteria and transfer-readiness identification reduced the time-to-transfer decision and increased the proportion of patients transferred to the floor on 6 Lpm HHF or greater. We achieved these outcomes without adverse effects, suggesting others can safely utilize our transfer criteria and process to facilitate the timely transfer of patients with viral bronchiolitis from the PICU to the general floor. However, further work is needed to translate study interventions into a more significant PICU LOS reduction.

## DISCLOSURE

The authors have no financial interest to declare in relation to the content of this article. This study was supported by the Agency for Healthcare Research and Quality (grant no. K08HS026512).

## ACKNOWLEDGEMENTS

The authors thank Dr. Dan Hyman, Dr. Jennifer Reese, Dr. Justin Beverly, Beth Wathen, and Dr. Victor Grazette for their executive sponsorship and support of this project. We also thank our front-line champions Erin Martin, Michelle Chiodini, Elizabeth Diaz, and Tiffany McCombs as well as Dr. Kathryn Squiers, and Lauren Rodgers for assistance with data collection, and Rebecca Colley for her expertise in process mapping and planning our intervention. Finally, we thank the full CHCO REST is Best Team and the University of Colorado IHQSE Certificate Training Program for the immense encouragement and support.

## Supplementary Material


