# Supplementary figures and images for: Reducing PICU-to-Floor Time-to-Transfer Decision in Critically Ill Bronchiolitis Patients using Quality Improvement Methodology
Source: Pediatr Qual Saf. 2022 Jan 21;7(1):e506. doi: 10.1097/pq9.0000000000000506 (PMC8782107; doi:10.1097/pq9.0000000000000506)

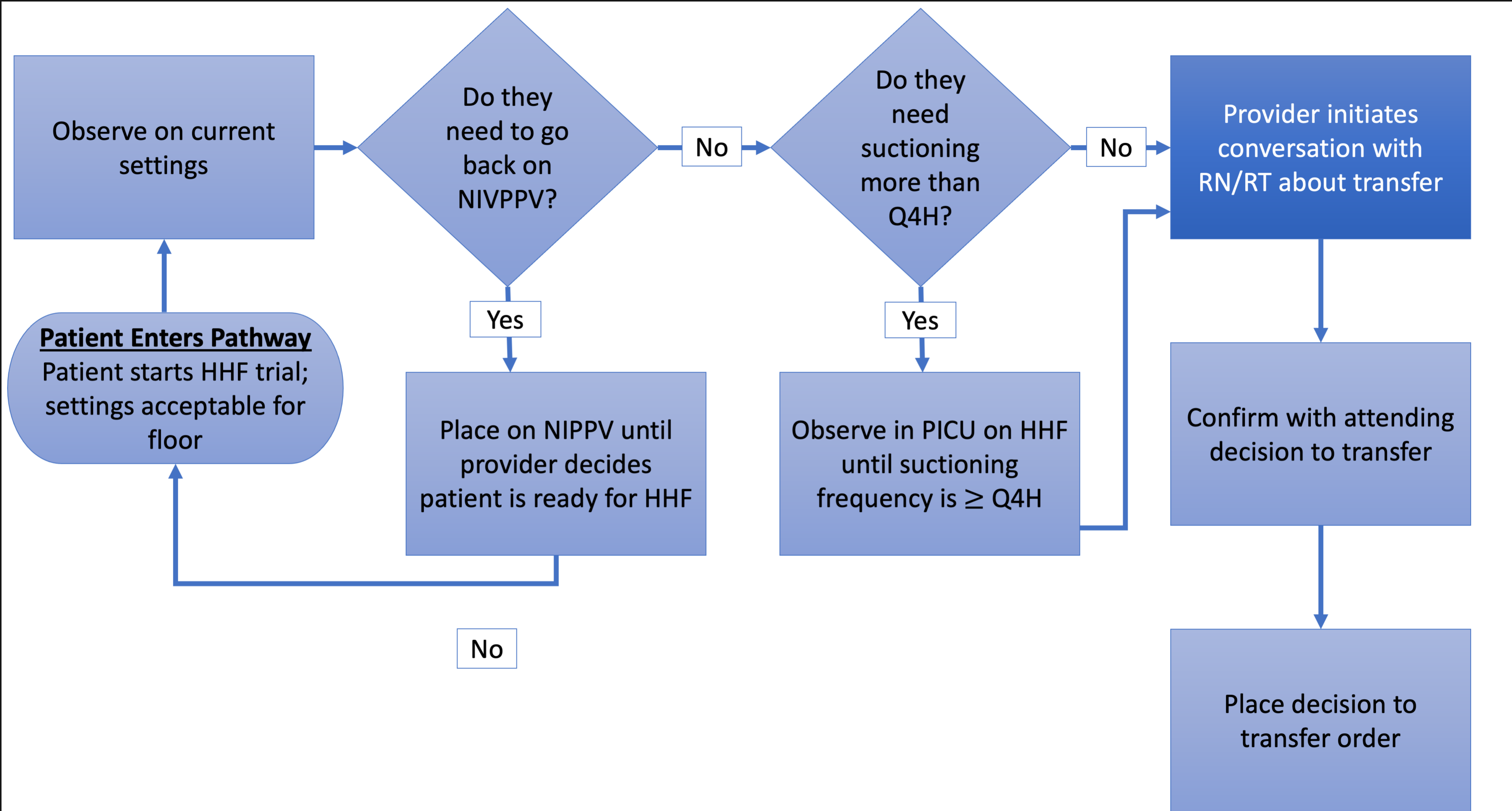

SCD, Figure 1. Pre-Intervention state process map

Supplement: Supplementary file 1 [file pqs-7-e506-s001.pdf]

SDC, Figure 4. Timeline of quality improvement group activities and key project dates

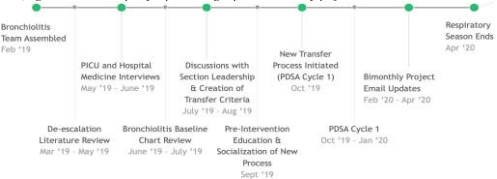

Supplement: Supplementary file 4 [file pqs-7-e506-s004.pdf]
