# Supplementary material for: Reducing PICU-to-Floor Time-to-Transfer Decision in Critically Ill Bronchiolitis Patients using Quality Improvement Methodology
Source: Pediatr Qual Saf. 2022 Jan 21;7(1):e506. doi: 10.1097/pq9.0000000000000506 (PMC8782107; doi:10.1097/pq9.0000000000000506)
Supplement: Supplementary file 3 [file pqs-7-e506-s003.pdf]

Is your patient with <u>bronchiolitis</u> ready for transfer to the floor?

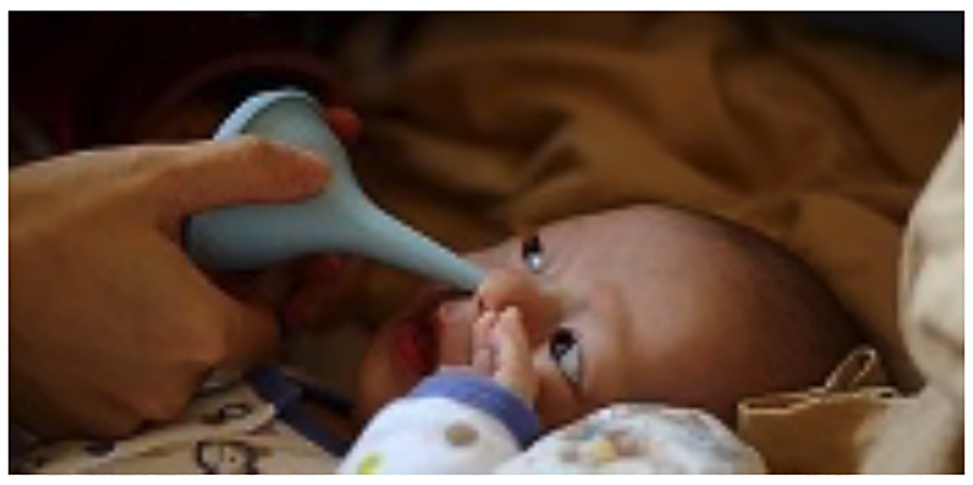

If your patient with viral bronchiolitis has been **on their maximum heated high flow rate (or lower) for 8 hours** and does not have any of the conditions listed below, please **contact the front-line provider** so they can perform an assessment to determine if the patient is ready for transfer*.

<u>Patients are not eligible for this pathway if any of the following are present:</u>

- Gestational age at birth < 36 weeks
- History of major cardiovascular disease
- History of chronic lung disease or baseline O_2/CPAP/BiPAP (*Asthma/Reactive Airway Disease and Pneumonia OK)*
- Chronic neurologic/neuromuscular disease**
- Intubated during this PICU admission
- Require suctioning more frequently than every 2 hours

Maximum heated high flow settings allowed on the floor:

Age of Patient	Maximum Floor (non-ICU) Flow Rate
1 month – 3 months	**6 liters per minute**, FiO2 $\leq$ 50%
3 months – 3 years	**8 liters per minute**, FiO2 $\leq$ 50%
3 years – 6 years	**10 liters per minute**, FiO2 $\leq$ 50%

*Interpretation of the 5 criteria is ultimately at the discretion of the ICU provider. When in doubt, the patient should be excluded / not considered for this transfer pathway. This form represents transfer *guidelines* only. The ultimate decision on transfer readiness is up to the ICU team.

***Examples of neurologic/neuromuscular disease include seizure disorder, cerebral palsy, traumatic brain injury, or other condition leading to significant developmental delay (e.g. trisomy 21).*

SDC, Figure 3. Educational flyer. Copies of the flyer were placed throughout the PICU and floor workspaces prior to project implementation to aid staff in identifying qualifying patients.
