# Supplementary material for: Reducing PICU-to-Floor Time-to-Transfer Decision in Critically Ill Bronchiolitis Patients using Quality Improvement Methodology
Source: Pediatr Qual Saf. 2022 Jan 21;7(1):e506. doi: 10.1097/pq9.0000000000000506 (PMC8782107; doi:10.1097/pq9.0000000000000506)
Supplement: Supplementary file 5 [file pqs-7-e506-s005.pdf]

Reducing PICU-to-Floor Time-to-Transfer Decision in Critically Ill Bronchiolitis Patients Using Quality Improvement Methodology:

Supplemental Digital Content

Supplemental Methods

Criteria for Special Cause Variation

As per the recommendations in Carey et al[1], special cause variation was considered to have been met when any of the following conditions occurred:

- When a single point falls outside of the control limit
- A trend of 6 or more values in a row steadily increasing or decreasing
- When two out of three successive values are: a) on the same side of the centerline, and b) more than 2 standard deviations from the centerline
- When eight or more successive values fall on the same side of the centerline

References

1. Carey RG, Stake LV. *Improving healthcare with control charts: basic and advanced SPC methods and case studies.* Quality Press; 2003.
