# Supplementary material for: Reducing PICU-to-Floor Time-to-Transfer Decision in Critically Ill Bronchiolitis Patients using Quality Improvement Methodology
Source: Pediatr Qual Saf. 2022 Jan 21;7(1):e506. doi: 10.1097/pq9.0000000000000506 (PMC8782107; doi:10.1097/pq9.0000000000000506)
Supplement: Supplementary file 6 [file pqs-7-e506-s006.pdf]

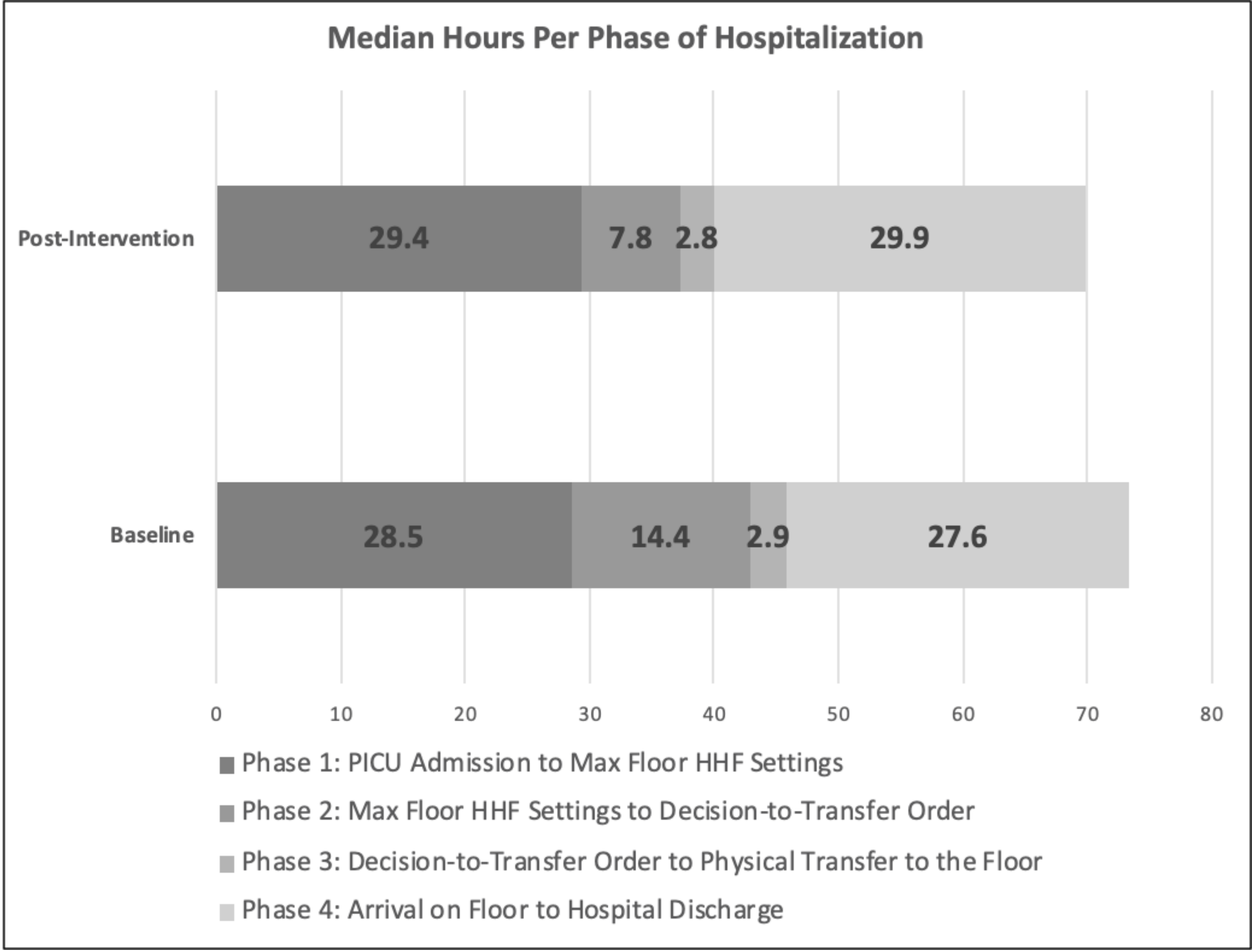

SDC, Figure 5. Median number of hours spent in each phase of a patient's hospitalization. Time on floor prior to PICU admission not shown. Note that because each phase value listed is the median value for that specific phase over all patients that the sum of phases 1, 2, and 3 does not equal the median PICU LOS.
Abbreviations: HHF = heated high flow nasal cannula
