# Supplementary material for: Reducing PICU-to-Floor Time-to-Transfer Decision in Critically Ill Bronchiolitis Patients using Quality Improvement Methodology
Source: Pediatr Qual Saf. 2022 Jan 21;7(1):e506. doi: 10.1097/pq9.0000000000000506 (PMC8782107; doi:10.1097/pq9.0000000000000506)
Supplement: Supplementary file 2 [file pqs-7-e506-s002.pdf]

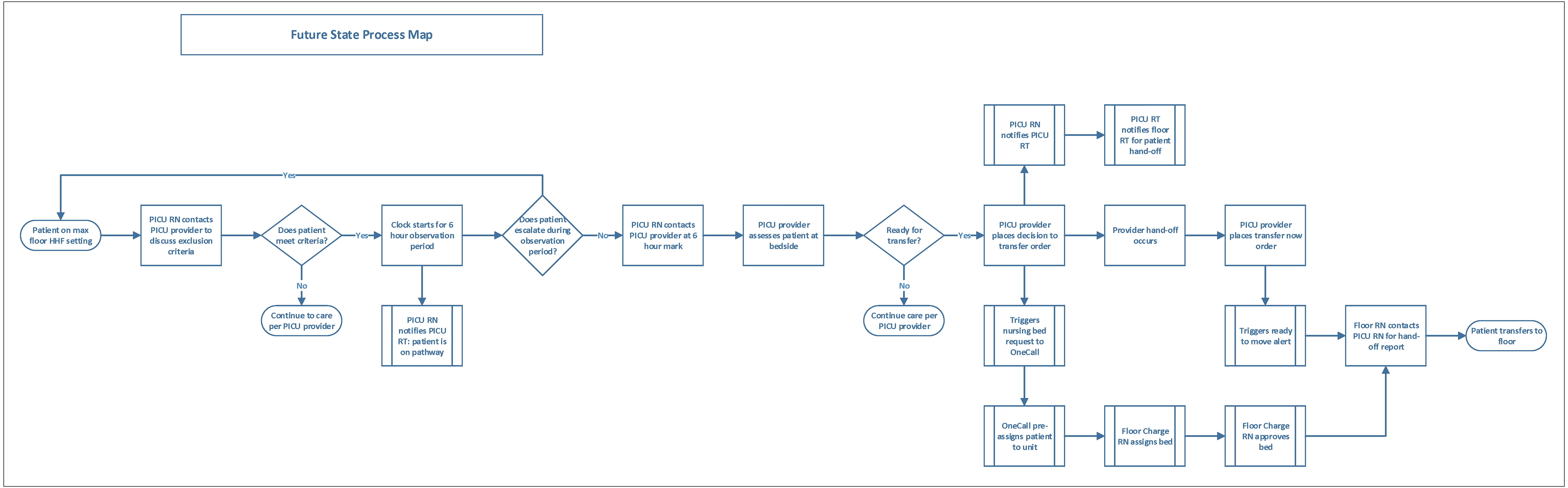

SCD, Figure 2. Future state process map.
